# 

**Published:** 1919-12-20

**Authors:** 


CHRISTMAS NUMBER.

### THE HOSPITAL

**The Modern Newspaper of Administrative Medicine and Institutional Life.**

Telegraphic Address: OSPEDALE, RAND, LONDON. Telephone Number: 2734 GERRARD.

No. 1750, Vol. LXVII. | Saturday, December 20th, 1919 | PRICE TWOPENCE

